# Latent profile analysis of depression in elderly patients with cardio- and cerebrovascular diseases in China– based on CLHLS data

**DOI:** 10.3389/fpsyt.2025.1556054

**Published:** 2025-03-21

**Authors:** Man Meng, Chen Zheng, Qi Hu

**Affiliations:** ^1^ First Affiliated Hospital of Jinzhou Medical University, Jinzhou, Liaoning, China; ^2^ Nursing Department, Shanxi Bethune Hospital, Taiyuan, Shanxi, China

**Keywords:** latent profile analysis, depression, elderly, cardio-and cerebrovascular, influencing factor

## Abstract

**Background:**

This study explored the depressive status of elderly patients with cardio- and cerebrovascular disease, using latent profile analysis to explore different profiles of depression. It also explored the factors influencing different profile of depression in patients with cardio- and cerebrovascular diseases to provide reference to healthcare workers to identify the high-risk group of anxiety and depression symptoms at an early stage.

**Methods:**

Data came from the Chinese Longitudinal Healthy Longevity Survey (CLHLS). In this study, we used latent profile analysis (LPA) to develop a latent profile model of elderly patients with cardio- and cerebrovascular disease combined with depression and to explore its influencing factors.

**Results:**

The 1890 study participants were divided into a low-level group (11%), a medium-level group (52%), and a high-level group (37%). The results of the univariate analysis showed statistically significant differences in the distribution of gender, age, co-residence, self-reported health, main source of financial support, marital status, diabetes, smoke, drank, exercise, level of anxiety, and IADL in the three profiles. Multiple logistic regression showed that good or fair self-reported health and exercise were associated with the low-level of depression; no spouse, and anxiety level were associated with moderately severe depressive conditions; and retirement wages, and local government or community predicted the appearance of low-level of depression compared to medium-level of depression.

## 1 Introduction

Due to increasing life expectancy and the persistence of low fertility, population ageing is accelerating globally and is one of the key demographic changes facing many countries. The global population of older persons aged 65 and over was projected to increase from more than 700 million in 2021 to 1 billion in 2050 (1). Population ageing and its accompanying health and social challenges are a global phenomenon and are no longer a problem faced by a few economically developed countries. China has been an ageing society since 2000 and became one of the world’s high-level ageing countries at the end of 2017. By 2010, there were about 111 million people over years of age in China, representing 8.2% of the total population. By 2050, China’s population aged 60 and older will reach 400 million, accounting for 26.9% of the world’s elderly population (2, 3). With the development of an ageing population, industrialization, urbanization and changes in lifestyle, the morbidity and mortality rates of acute infectious and parasitic diseases have been declining, and chronic non-communicable diseases have become the main cause of death and the burden of disease for Chinese residents (4–6). The elderly, whose physiological functions are declining and their ability to resist diseases is decreasing, belong to a group of people with a high prevalence rate, characterized by a large proportion of chronic diseases and a high rate of disability. The further aggravation of the degree of aging has led to a significant increase in the prevalence of chronic diseases, dysfunction and risk of incapacitation, which is an important cause of the decline in the ability of the elderly to take care of themselves, the occurrence of diseases and death (7). Not only does it bring suffering to the physical and mental health of older persons, but it also increases the burden of old age on families and society, and has become a major public health problem affecting the country’s economic development (8, 9). Cardiovascular and cerebrovascular diseases account for the largest proportion of deaths due to chronic non-communicable diseases and are the number one cause of death worldwide (10). Cardiovascular and cerebrovascular diseases is a collective term for cardiovascular and cerebrovascular diseases, which refers to ischemic or hemorrhagic diseases of the heart, brain, and tissues of the whole body due to hyperlipidemia, blood viscosity, atherosclerosis, and high blood pressure (11). Due to its high morbidity, disability, mortality, recurrence, and multiple complications, it has become the primary disease that seriously affects the health level and quality of life of the elderly. It has long been a major disease burden worldwide (12, 13). In China, with the aging of the population and changes in lifestyle and environmental factors, cardiovascular and cerebrovascular diseases have become one of the serious public health challenges facing China.

In order to improve the health and quality of life of the elderly, the World Health Organization (WHO) has defined healthy ageing as a process of maintaining functional ability to enable wellbeing in older age (14). The level of individual health of the elderly is categorized into three dimensions: Body health, Psychological health and Social adaptation (15). Healthy ageing is not only reflected in the good physical condition and material wealth of the elderly, but also in the good psychological state of the elderly (16, 17). Psychological health refers to a healthy personality with positive cognitive, affective, and volitional behaviors, and the ability to maintain positive attitudes and behaviors. And depression, a common mental illness that seriously affects physical and mental health, the symptoms are mainly depressed mood, but also includes slow thinking, poor mood, pessimistic attitude, lack of passion and vitality, reduced will activity, poor sleep quality and other physical symptoms, and in severe cases, even suicidal behavior (18). Depression is receiving increasing attention as an important indicator of mental health (19). Physical health status is a major correlate of depressed mood in the elderly (20). Cardiovascular and cerebrovascular diseases, as diseases with high morbidity and mortality in the elderly population, tend to have a sudden onset and a long treatment time. Elderly people are often in a stress reaction due to the uncertainty of the disease, which increases the patient’s psychological burden resulting in depressive symptoms, which seriously affects the physical and mental health and quality of life of the elderly population (21).Studies showed that cardio- and cerebrovascular disease plays a causal role in the development of depression in the elderly due to factors such as underlying biological and psychosocial pathogenic mechanisms (22, 23). The American Heart Association and the European Society of Cardiology document that depression is a prognostic factor for cardio- and cerebrovascular disease (24, 25). Depression not only seriously affects the physical health of elderly patients with cardiovascular and cerebrovascular diseases, aggravates their incapacitating state, and reduces their quality of life, but also increases the risk of cognitive impairment and leads to the formation of suicidal ideation. These harms will not only reduce the life satisfaction of the elderly, but will also bring a heavy economic burden to the family and even the society.

Previous research on depression in elderly patients with cardio- and cerebrovascular disease focused on different genders, different regions, and the relationship between depression and other factors (25–27). Most of these studies judged the depressive status of older adults based on total questionnaire scores and thresholds, and their results reflected overall averages and ignored individual differences. Latent profile analysis (LPA) was an individual-center analysis to determine the classification of latent depression profile in elderly cardio- and cerebrovascular patients, to understand the distributional characteristics of each type of person, and to explore individual heterogeneity (28). LPA was based on the estimation of objective statistical indicators from model fitting and was achieved by using latent categorical variables to explain associations between external continuous variables, thus achieving local independence between the display variables (29). No studies have been conducted to investigate the subtypes of depression in elderly patients with cardiovascular and cerebrovascular diseases. Based on this, the present study proactively used LPA to identify types of depression in elderly patients with cardio- and cerebrovascular disease, with the aim of providing physicians and public health managers with an accurate and objective identification of depression subtypes in this population, and providing a scientific basis for implementing targeted psychological interventions.

## 2 Materials and methods

### 2.1 Sample

**Figure 1:**
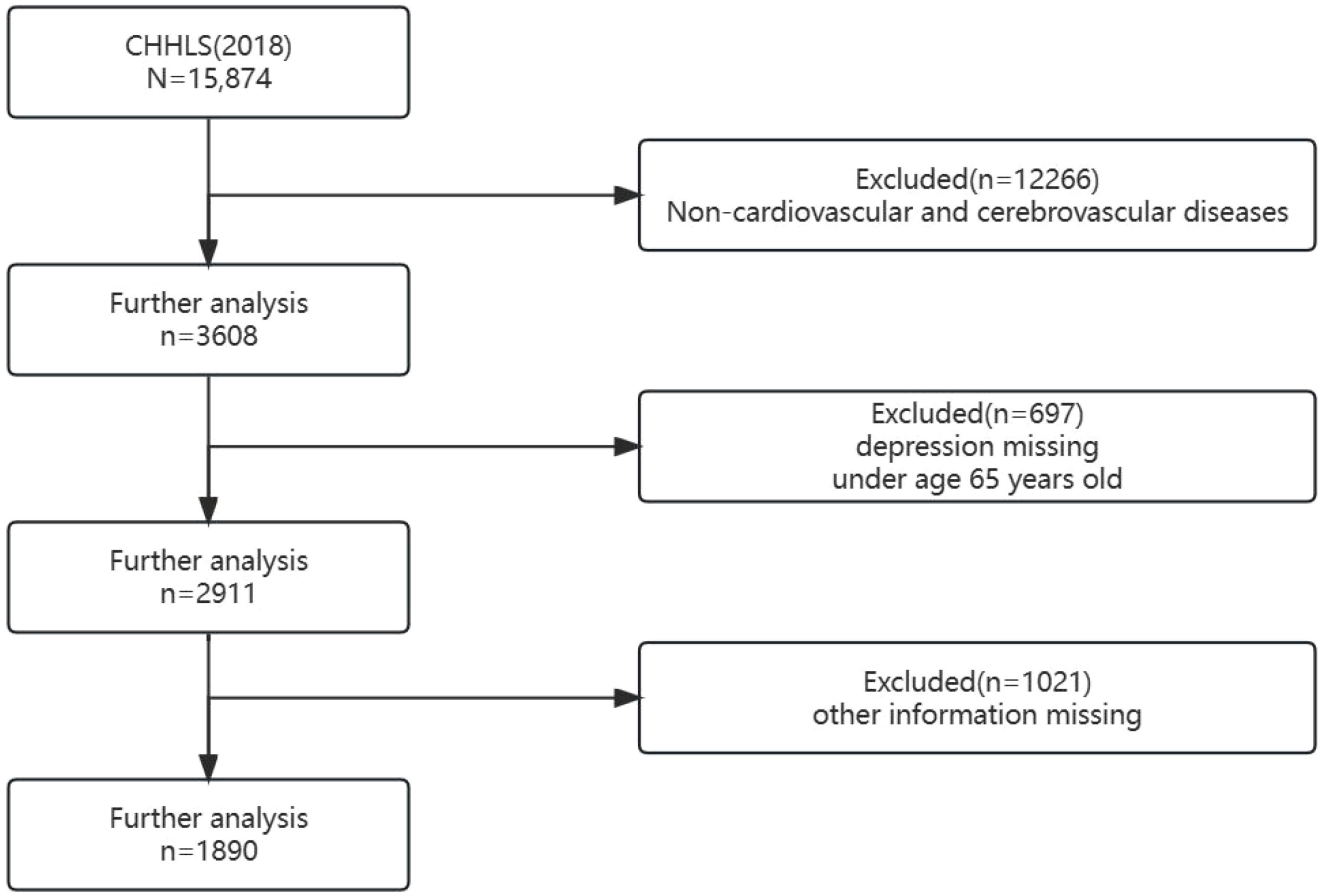
Flow diagram of the selection of sample.

Data for this study came from the China Longitudinal Healthy Lifespan Survey (CLHLS) (https://opendata.pku.edu.cn/), jointly organized by the Center for Healthy Ageing and Development at Peking University and the National Development Research Institute. The survey conducted eight follow-up surveys of China’s elderly population aged 65 and older between 1998 and 2018. The research used a multistage stratified sampling method to conduct household interviews with older people 65 and older, with the sample covering 23 provinces, municipalities and autonomous regions in China, and surveying a total of 113,000 people. All study participants signed an informed consent form. The researcher recorded and double-checked the recordings throughout the study to ensure the authenticity of the sample and to assess the quality of the data. The survey included individual microdata on the physical and mental health of the elderly, behavioral patterns, socioeconomic status, family structure, intergenerational relationships, and care provision and costs, which was highly representative of research on issues related to the elderly population of China and was generally accepted by scholars at home and abroad. This paper used data from the 2018 cross-section published by the Open Research Data Platform of Peking University (Peking University Center for Healthy Aging and Development Studies, 2020). In terms of data processing, 15,874 samples were obtained from the original data, and 1,890 valid samples were obtained after excluding samples under the age of 65, samples with missing values of the core variables, and samples that answered “don’t know, refused to answer” (**Figure 1**).

### 2.2 Measurement

#### 2.2.1 General demographic information

We choose the following variables as information on demographic characteristics of the study population, including gender, age, current residential, co-residence, self-reported health, smoke, drink, exercise, education level, marital status, health care payment method, source of financial support, serious medical condition, hypertension, hyperlipidemia, and diabetes. The variable assignments were shown in **Table 1**, with anxiety and IADL as continuous variables (**Table 1**).

**Table 1:** Multivariate logistic regression analysis of different potential factors in elderly patients with cardiovascular and cerebrovascular.

Variables	Low VS High			Low VS Medium			Medium VS High		
	OR	95%CI	*P*	OR	95%CI	*P*	OR	95%CI	*P*
Gender									
Male	0.765	0.453-1.292	0.317	0.946	0.722-1.240	0.869	0.809	0.501-1.305	0.385
Age									
65-75	2.098	0.831-5.297	0.117	1.278	0.793-2.508	0.314	1.642	0.702-3.843	0.253
75-85	2.386	1.051-5.415	0.038	1.599	1.051-2.432	0.028	1.492	0.701-3.174	0.299
85-95	1.911	0.887-4.117	0.098	1.621	1.104-2.382	0.014	1.179	0.579-2.399	0.650
co-residence of interviewee									
With household member(s)	0.576	0.240-1.382	0.216	0.574	0.339-0.971	0.038	1.004	0.472-2.133	0.992
Alone	0.719	0.275-1.881	0.505	0.591	0.331-1.506	0.076	1.216	0.531-2.787	0.644
Self-rated health									
Good	0.089	0.048-1.164	<0.001	0.373	0.272-0.512	<0.001	0.237	0.135-0.417	<0.001
Fair	0.038	0.193-0.492	<0.001	0.730	0.537-0.991	0.044	0.423	0.285-0.627	<0.001
Main source of financial support									
retirement wages	2.798	0.167-1.043	0.043	1.167	0.718-1.897	0.553	2.379	0.958-5.996	0.062
Family	1.371	0.319-2.002	0.538	1.095	0.669-1.792	0.719	1.252	0.499-3.138	0.632
local government or community	3.565	0.146-1.066	0.024	1.408	0.777-2.551	0.260	2.532	0.938-6.835	0.067
work by self	0.718	0.541-8.837	0.665	1.569	0.822-2.996	0.172	0.458	0.113-1.850	0.273
Marital status									
No spouse	1.432	0.888-2.309	0.140	1.925	1.139-3.253	0.037	1.344	1.018-1.775	0.037
Hyperglycemia									
Yes	1.117	0.679-1.837	0.973	1.109	0.839-1.465	0.468	1.008	0.645-1.574	0.973
Smoke									
Yes	0.745	0.514-1.609	0.910	0.860	0.650-1.138	0.291	1.058	0.623-1.796	0.836
Drink									
Yes	0.765	0.425-1.376	0.371	0.904	0.680-1.202	0.487	0.846	0.490-1.459	0.548
Exercise									
Yes	0.363	0.223-0.589	<0.001	0.808	0.641-1.018	0.071	0.449	0.286-0.703	<0.001
Anxiety	2.179	0.972-1.063	<0.001	1.531	1.407-1.667	<0.001	1.423	1.345-1.505	<0.001
IADL	1.017	0.972-1.063	0.468	1.000	0.975-1.025	0.988	1.017	0.977-1.059	0.414

#### 2.2.2 Depression

The Center for epidemiological studies depression scale (CES-D-10) was used to assess the degree of depression in elderly patients with cardio-cerebrovascular disease (30). The CES-D-10 scale consisted of ten items consisting of seven negative questions and three positive questions. The answers to the negative questions were scored in the following order: 0, 1, 2, and 3, while the positive questions were scored in reverse order. The total score was 0-30. The frequency of depression-related symptoms or behaviors in the past week was measured primarily. Depressive symptom burden was defined by scale scores: individuals were defined as having a high depressive symptom burden (HDSB) if they met a baseline CESD-10 score of ≥10, and vice versa as having a low depressive symptom burden (LDSB). Studies showed that CESD-10 was proven to be a valid and reliable tool to detect depressive symptoms in the elderly population (31). The Cronbach’s value of the scale in this study was 0.822.

#### 2.2.3 Anxiety

The CLHLS used the Generalized Anxiety Disorder Scale (GAD-7) to assess the frequency of anxiety symptoms in the lives of patients over a two-week period. The scale consisted of seven items, with response categories including “not at all”, “a few days”, “more than half the days”, and “almost every day”. “ which correspond to scores of 0, 1, 2, and 3, respectively. The scores for the seven items were added together to form the GAD-7 questionnaire score. The total GAD-7 score ranges from 0 to 21. Spitzer et al. used an internal consistency coefficient Cronbach alpha coefficient of 0.92 for GAD-7 in the primary care center attending population (27). The Cronbach coefficient for anxiety in the present study was 0.922.

#### 2.2.4 Instrumental activities of daily living scale

The use of the Instrumental activities of daily living scale (IADL) in the CLHLS was used to assess the health status related to socialization skills among older Chinese people living in the community (32). The IADL consisted of eight main items, including visiting neighbors independently; shopping independently; cooking independently; washing clothes independently; lifting 5 kg independently; walking 2 km continuously; squatting 3 times continuously; and traveling independently by transport. In the CLHLS survey, the response options for the eight questions were 3 for “Yes, very restricted”, 2 for “Yes, somewhat restricted”, and 1 for “Not restricted”. “. The higher the score, the worse the IADL. The scale had good reliability and was widely used in the Chinese elderly population (33). The Cronbach’s coefficient of the IADL scale in this study was 0.947.

### 2.3 Statistical analysis

#### 2.3.1 Common method deviation test

Data needed to be checked for the common method deviation test as the data was collected by participants using self-reported methods with a large number of questionnaire items, which could confound the findings and potentially mislead the conclusions, generating systematic errors. Data were examined in this study using the Harman single factor method (34). All scale items were placed into the same unrotated exploratory factor analysis, and there was no common method bias if at least two common factors could be extracted from the analysis, and the first one explained no more than 40% of the variance.

#### 2.3.2 Descriptive analysis

SPSS 26.0 was used in this study for data collation and analysis. Data were tested for normality, and measurement data conforming to the normal distribution were expressed as mean ± standard deviation (M ± SD), the nonnormal distribution was expressed as median (M) and quartiles (IQR), and categorical data were expressed as frequency and percentage. Some of the variable definitions and codes are shown in **Table 2**.

**Table 2:** Case of variable assignment.

Variable	Assignment mode
Gender	Male=1; Female=2
Age	65-75 = 1; 76-85 = 2;86-95 = 3;>96 = 4
residence	City=1;=2;Rural=3
co-residence of interviewee	With household member(s) =1;Alone=2;In an institution=3
Self-rated health	Good=1;Fair=2;Bad=3
Education level	None1;0-6 = 2;≥7 = 3
Main source of financial support	retirement wages=1;Family=2;local government or community=3;work by self=4;others=5
Marital status	No spouse =1;Have a spouse=2;
Medical payment method	Medical insurance=1;Family=2;Others=3
Serious illness	None=1; ≥1 = 2
Hypertension	Yes=1; No=2
Hyperglycemia	Yes=1; No=2
Hyperlipemia	Yes=1; No=2
Smoke	Yes=1; No=2
Drink	Yes=1; No=2
Exercise	Yes=1; No=2
Anxiety	Measured value
BADL	Measured value

#### 2.3.3 Latent profile analysis

In this study, the CES-D-10 score was used as an observational indicator and the data were analyzed by potential profiling using Mplus software. Model fitting was performed starting from the initial model (assuming the existence of one potential category) and then sequentially increasing the number of potential categories. Based on Akaike information criteria (AIC), Bayesian information criterion (BIC), entropy, Lo-Mendell-Rubin likelihood ratio test (LMR), Bootstrapped likeli-hood ratio test (BLRT) and other fitting metrics were comprehensively evaluated. The optimal model was selected by taking into account the actual situation and the practical significance of each type of model.

#### 2.3.4 Single factor and multi-factor analysis

The results of the latent profile analysis category analysis were used as the dependent variable, and the chi-square test and the Kruskal-Wallis test were used for comparisons between groups. Statistically significant general information was selected as independent variables, and multiple logistic regression was used to analyze the influencing factors of different profiles of depression in elderly cardio- and cerebrovascular patients. *P*<0.05 was taken as statistically significant difference.

## 3 Results

### 3.1 Common method deviation test

The results of the study showed that four common factors could be extracted after putting all items into the model, and the maximum variance of the common factor explained was 21.92%, which was less than 40%, indicating that there was no significant bias of the common method present in this study.

### 3.2 Demographic analysis

A total of 1890 elderly people over 65 years of age were included in this study. Of these, 861 were male, accounting for approximately 45.6% of the total sample. The age group was mainly concentrated between 76-85 years old, and most of the patients chose to live with family members, with a predominantly nonformal education. The primary financial source for most elderly patients with cardio- and cerebrovascular disease was their retirement pay. Medical insurance was chosen to cover medical expenses. Additional information was shown in **Table 3**.

**Table 3:** A univariate analysis of potential categories of depression in elderly patients with cardiovascular and cerebrovascular.

Characteristics	N(%)	Low-level (N=215)	Medium-level (N=978)	High-level (N=697)	χ^2^/H	*P*
Gender					χ^2^ =22.605	<0.001
Male	861 (45.6)	74	427	360		
Female	1029 (54.4)	141	551	337		
Age						
65-75	537 (28.4)	58	264	215	χ2 = 13.879	0.031
76-85	675 (35.7)	90	362	223		
86-95	464 (24.6)	47	252	165		
>96	214 (11.3)	20	100	94		
residence						
City	752 (39.8)	92	380	280	χ2 = 1.617	0.806
Town	518 (27.4)	58	267	193		
Rural	620 (32.8)	65	331	224		
co-residence of interviewee						
With household member(s)	1488 (78.7)	156	757	575	χ2 = 14.485	0.006
Alone	301 (15.9)	44	159	98		
In an institution	101 (5.3)	15	62	24		
Self-rated health					χ2 = 250.986	<0.001
Good	642 (34.0)	24	276	342		
Fair	794 (42.0)	65	460	269		
Bad	454 (24.0)	126	242	86		
Education level					χ2 = 9.506	0.05
None	691 (36.6)	92	372	227		
0-6	618 (32.7)	61	313	244		
≥7	581 (30.7)	62	293	226		
Main source of financial support					χ2 = 20.150	0.010
retirement wages	846 (44.8)	89	424	333		
Family	664 (35.1)	77	350	237		
local government or community	169 (8.9)	33	87	49		
work by self	109 (5.8)	6	64	39		
others	102 (5.4)	10	53	39		
Marital status					χ2 = 6.115	0.047
No spouse	930 (49.2)	97	465	368		
Have a spouse	960 (50.8)	118	513	329		
Medical payment method					χ2 = 4.415	0.353
Medical incurance	1188 (62.9)	130	625	433		
Family	665 (35.2)	82	338	245		
Others	37 (2.0)	3	15	19		
Serious illness					χ2 = 3.767	0.152
None	1037 (54.9)	106	535	396		
≥1	853 (45.1)	109	443	301		
Hypertension						
Yes	1181 (62.5)	143	608	430	χ2 = 1.716	0.424
No	709 (37.5)	72	370	267		
Diabetes					χ2 = 7.835	0.020
Yes	337 (19.9)	55	202	120		
No	1513 (80.1)	160	776	557		
Hyperlipemia					χ2 = 2.945	0.229
Yes	281 (14.9)	40	137	104		
No	1069 (85.1)	175	841	593		
Smoke					χ2 = 14.125	0.001
Yes	182 (30.8)	51	283	248		
No	1308 (69.2)	164	695	449		
Drink					χ2 = 9.558	0.008
Yes	462 (24.4)	41	225	196		
No	1428 (75.6)	174	753	501		
Exercise					χ2 = 50.149	<0.001
Yes	773 (38.8)	42	369	322		
No	1157 (61.2)	173	609	375		
Anxiety[M(IQR)]	0(2)	215	978	697	H=523.139	<0.001
BADL[M(IQR)]	11(10)	215	978	697	H=40.009	<0.001

### 3.3 Results of latent profile analysis

Latent profiles analysis was conducted on the depression status of 1890 participants. One to five potential profile models were established sequentially, and the results of the fit indicators for each model are shown in **Table 4**. In the fitted 1-5 models: (1) the values of AIC, BIC, aBIC gradually decreased in the process of increasing the number of profiles from 1 to 5, the more the number of profiles, the better the fit, in this step excluded the models with 1 and 2 profiles; (2) when the number of model profiles were 5, the *P*-values of LMR and BLRT were 0.4281 and 1, respectively, with P>0.05, and were excluded; (3) Entropy > 0.8 for both the 3-profile model and the 4-profile model, but in the 4-profile model and the presence of a subgroup with only 5% of the total number of people, more valid information was dispersed and interpretability was poor. Therefore, the model with a profile number of 3 was finally chosen as the optimal potential profile model (**Table 4**).

**Table 4:** Indicators for latent profile of depression in elderly patients with cardiovascular and cerebrovascular.

Profile	K	Likelihood	AIC	BIC	aBIC	Entropy	LMR(P)	BLRT (P)	Proportion
1	20	-27494.240	55028.480	55139.366	55075.827	–	–	–	–
2	31	-25763.942	51589.833	51761.758	51663.271	0.792	0.0000	0.0000	0.42/0.58
3	42	-25150.380	50384.760	50617.622	50484.188	0.835	0.0000	0.0000	0.11/0.52/0.37
4	53	-24937.482	49980.965	50274.814	50106.433	0.858	0.0000	0.0000	0.11/0.50/0.34/0.05
5	64	-24763.371	49654.742	50009.579	49806.251	0.791	0.4281	1	0.07/0.30/0.30/0.08/0.24

The average probability of attribution was calculated for each potential profile in the 3-profile model to determine the precision of the classification results. The mean probability of attribution for profile 1 was 0.918, for profile 2 was 0.922, and for profile 3 was 0.929, all of which were >0.9 indicating that the model fit was reliable when the number of profiles was three (**Table 5**).

**Table 5:** Attribution probabilities for each latent profile of subjects.

Class	Profile 1	Profile 2	Profile 3
Profile 1	0.918	0.082	0.000
Profile 2	0.022	0.922	0.057
Profile 3	0.000	0.071	0.929

### 3.4 Naming of latent profile

**Figure 2:**
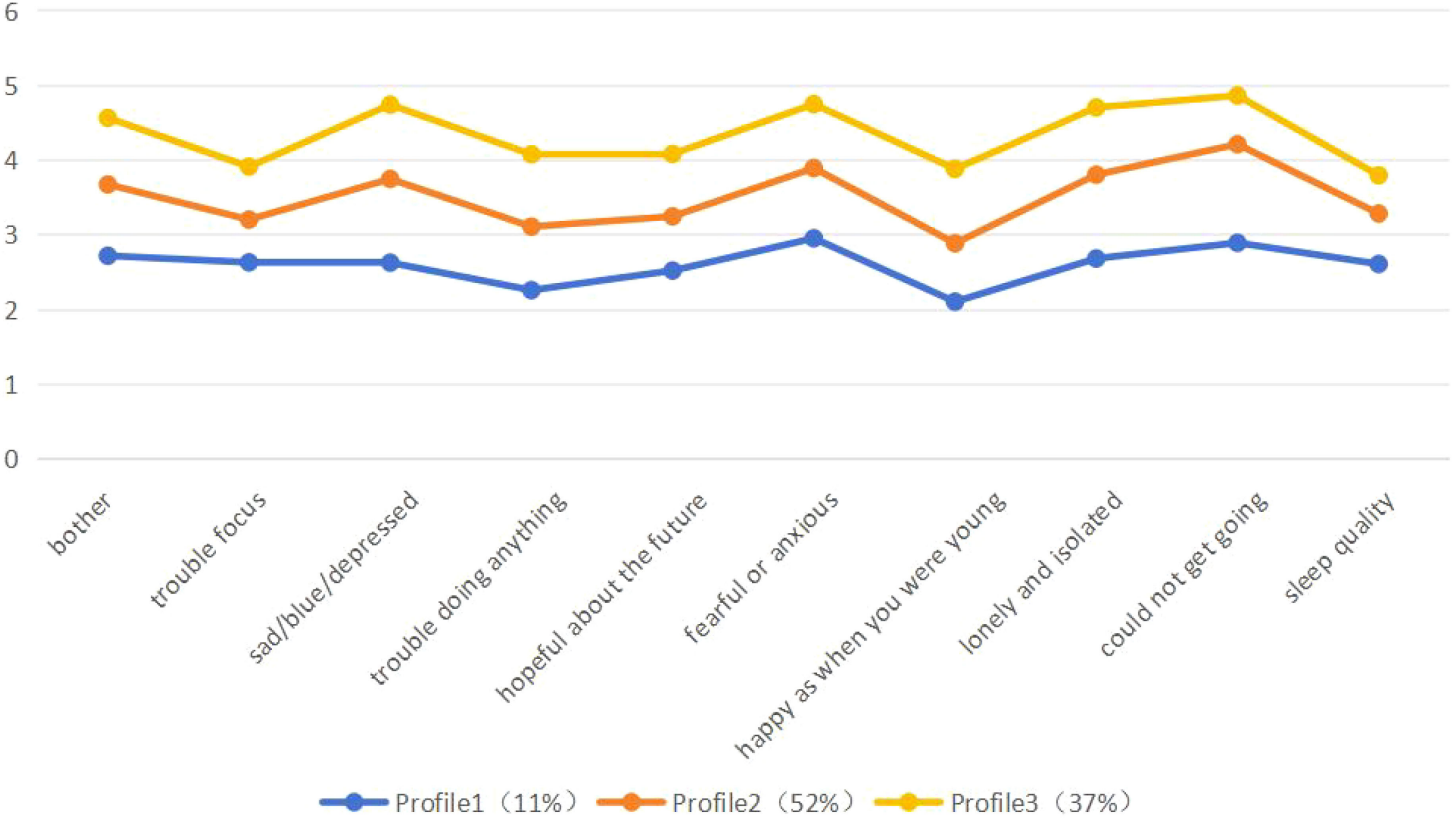
Latent profile model of depression in elderly people with cardio- and cerebrovascular diseases.

The potential profile of depression in elderly patients with cardio- and cerebrovascular disease is shown in **Figure 1**. Profile 1, profile 2 and profile 3 depression scores increased in that order. Profile 1 had a low score of 11 percent on item 7, “Do you feel as happy as you are when you are young?”, and based on the characteristics of its score, the category was named “low level”. Profile 3 scored significantly higher than categories 1 and 2, at 37%, and was named “high level”. Profile 2 was scored between profiles 1 and 3, accounting for 52% of the total, and based on the characteristics of its scores, the profile was named “medium level” (**Figure 2**).

### 3.5 Inter-profile characteristic differences

Differences in demographic and sociological information, anxiety and IADL were compared using potential depression profiles in study participants as subgroup variables. *P* < 0.05 indicated a statistically significant difference. The results showed statistically significant differences in the distribution of gender, age, co-residence, self-reported health, source of financial support, marital status, diabetes, smoke, drink, exercise, anxiety level, and instrumental daily activity capacity in the three profiles. (*P* < 0.05) See **Table 3** for details.

### 3.6 Multinomial logistic regression of depression profiles

General demographic information with statistically significant differences in univariate analyzes and level of anxiety and degree of instrumental daily living ability were regression analyses with the underlying category of depression as the independent variable. Comparisons between groups were made using low and medium levels as reference groups. The results showed that gender, diabetes, smoke, drink and IADL did not differ significantly between the three profiles. Age, self-reported health, no spouse, anxiety and co-residence of interviewee predicted the moderate depression level pattern in the comparison of the low-level group with the medium-level group; self-reported health, no spouse, anxiety and exercise predicted the profile of the high-level group when comparing the low and medium level groups with the high-level group, respectively. See **Table 1** for details.

## 4 Discussion

This study used latent profile analysis to explore the heterogeneity of depression in elderly patients with cardio- and cerebrovascular based on various fitted indicators with practical implications. The results showed significant profile characteristics of depression in elderly cardio- and cerebrovascular patients and good fit of the indicators of the model when classified into three profiles, suggesting heterogeneity of depression among elderly individuals (17). Most of the previous studies on the level of depression in the elderly were based on the level of scale scores or critical values to determine their depression, but the present study used the”individual-centered”latent profile analysis to fit the potential categories of depression in Chinese elderly cardiovascular and cerebrovascular disease patients in a relatively large sample, identified the heterogeneous differences in depression among individuals, which provides a reference for the development of precise preventive and interventional measures for elderly patients with cardiovascular disease.

11% of elderly patients with cardio- and cerebrovascular disease were classified in the “low-level”, the smallest proportion, and scored low on all items. However, in item 6, “are you nervous and scared?” scored significantly higher than the other items, which may be due to the fact that after the occurrence of cardio- and cerebrovascular diseases, the patients were accompanied by varying degrees of anxiety and depression, which affected their physical and mental health, as well as their quality of life to varying degrees (35). For this group of people, although the degree of depression is mild, we should closely observe the mental health status of the patients and provide timely psychological counseling to avoid evolving towards a state of moderate to severe depression.

“Medium-level” has the highest share of the three profiles, accounting for about 52% of the total. The highest mean score on the scale was for item 9, “Do you feel unable to get on with your life”. This may be related to the fact that due to the severity and suddenness of cardiovascular and cerebrovascular diseases; it reduced the quality of life of elderly patients and increased their tension and psychological burden. Meanwhile, cardiovascular and cerebrovascular diseases required long-term medication and high treatment costs, which increased the financial pressure of elderly patients, which in turn led to depressive symptoms (36). Healthcare workers should focus on positive psychological interventions and health promotion by eliminating patients adverse perceptions of the disease, mobilizing their intrinsic positive resources, promoting patients’ adaptation to the disease, and alleviating their sense of uncertainty about the disease (37).

The “high-level” profile accounted for 37% of the total sample, and their scores on all items were significantly higher than those of the “low-level” and “medium-level “profile, suggesting that the overall level of depression in this profile was high. Under the influence of factors such as illness, loss of function, inability to take care of themselves and limited social interaction, elderly patients with cardio- and cerebrovascular were more likely to suffer from depression and other negative psychological conditions. In addition to this, most elderly people did not know about depression, and most were divorced or widowed, resulting in a lack of companionship and psychological comfort, leading to depression that cannot be resolved or for which they did not take the initiative to seek help, thus affecting quality of life and resulting in moderate or severe depression (38). Although older adults may have shown depressive tendencies, not receiving timely and effective mental health interventions and treatments could eventually lead to the exacerbation of depressive problems (39). Therefore, in the future prevention and control of depression in the elderly, we should focus on the mental health status of this group of people, actively conduct mental health lectures, help elderly patients with cardiovascular and cerebrovascular diseases to perform positive self-adjustment, cultivate the patient’s self-management ability in terms of alleviating symptomatic distress and promoting the enhancement of psychological resilience, as well as improving the adaptability of the disease, and eliminating the depressive mood of elderly patients (40).

In this study, factors such as living source situation and economic level were strongly associated with the occurrence of depressive symptoms in Chinese elderly patients with cardiovascular disease. In addition, age, marital status, residential situation and Self-reported health were also shown to be important factors influencing the occurrence of depressive symptoms. This study demonstrated that age was a risk factor for depression in elderly patients with cardio- and cerebrovascular disease, and older adults were more likely to be classified into the moderate-to-high depression level profile, which was consistent with the study by Miller et al (41). With age, the physiological functions of the elderly decline, and most of the cardiovascular and cerebrovascular diseases are accompanied by a decline in the ability to take care of themselves, due to poorer health, the probability of complications will also increase, the course of the disease is relatively long, the elderly are prone to worry about the disease and even anxiety (42). In addition, the social role of the elderly will also change after entering old age, and their psychological functions will also enter a decline period. The limited communication between the elderly and the outside world, and the decline in the degree of social participation will have a certain impact on the psychology of the elderly, which will make their perception of aging more obvious, thus leading to an increase in the risk of depression, and ultimately, the emergence of depressive symptoms (43).

Financial resources were the key factor influencing the level of depression in older people when comparing the low-level profile with the medium-level profile. Elderly patients with cardio- and cerebrovascular diseases required long-term medication and regular check-ups, which meant that patients needed adequate financial support to meet their daily needs as well as medical expense (44). When older people cannot secure basic daily needs and medical needs without regular daily financial resources, negative emotions, such as feelings of worthlessness may be heightened, leading to more negative depressive moods. Therefore, it is necessary to provide elderly cardiovascular and cerebrovascular patients with adequate financial protection and to pay attention to and care for the physical and mental health of the elderly, especially from the family to the government to the social level.

The results suggested that self-reported health status similarly affects the ease level of older adults. Self-reported health negatively predicted depressive symptoms, and better self-reported health was associated with lower symptom scores of depressives, consistently with Boima et al (45, 46). The greater the number of chronic conditions, the worse the self-reported health of older people and the more severe the depressive symptoms (42). The community could strengthen health education, raise awareness of self-health management among the elderly, provided long-term medical and nursing care services for the elderly at different levels, help the elderly establish a correct outlook on health, improve their self-assessment of health, and reduce the appearance and development of depressive symptoms.

Compared to the intermediate level subgroup, we found that marital status and residential status were also influential factors for depression in elderly patients with cardio- and cerebrovascular disease. Patients lived alone because they were unmarried, divorced, widowed, etc. They lacked family care and social support, and their spiritual needs could not be met, making them more prone to loneliness (47). The relationship between older people and their spouses was relatively stable and influential throughout the life cycle. Widowhood in old age not only increased psychological morbidity in patients, but also increased mortality from cardiovascular and cerebrovascular diseases (48). The Srivastava study showed that widowed older people living alone were 56% more likely to suffer from depression than married older people living together and 13.6% of older people over 65 years of who lived alone were depressed (49). Elderly patients with cardiovascular and cerebrovascular diseases required long-term medication to treat or slow the onset of disease or complications. Elderly people living alone not only need to take care of their own bodies but also to ensure their own quality of life and medication safety, the lack of family members to accompany them makes their negative emotions cannot be solved in a timely manner, this way of life makes them more prone to depression. The emotional and spiritual comfort of the family could not be replaced by material and financial means, so more attention was paid to the mental health state of this group of people, and timely psychological interventions were carried out to communicate emotionally with the elderly and reduce the incidence of depression.

Research found that exercise not only improves physical functioning but also enhances self-efficacy and also reduces depression to some extent (50, 51). Exercise therapy was widely regarded as an intervention method that was beneficial to the physical and mental health of patients with depression and of great practical value (52). Furthermore, low-cost exercise therapy, easy to implement, the advantages of scientifically designed exercise programs can effectively guarantee the effectiveness of therapy and reduced depression recurrence rate of the subjects (53).

In our study, anxiety differentiated significantly between these three conditions. Elderly patients with cardiovascular and cerebrovascular diseases must face problems such as chronic illnesses, movement disorders, and physical disabilities, in addition to the fact that they have to stay at home for a longer period of time after retirement, and their social contacts are narrowed down, which can also cause anxiety among the elderly (54, 55). Physical problems, mobility limitations, and dependence on others lead to anxiety and fear, and then to depression in older people. Giving good social support to the elderly at the social and family levels keeps them optimistic, thus reducing the emergence of anxiety and depression, and together we can maintain and promote the physical and mental health of the elderly.

With the deterioration of bodily functions due to ageing and the impact of complications from cardiovascular and cerebrovascular diseases, the elderly are often faced with the plight of limited physical mobility and loss of social functioning, which seriously affects their mental health. The probability of depression is higher among the depressed elderly group, with the disabled elderly being more likely to be depressed (56, 57). Under current circumstances, diversified forms of care services should be provided to disabled elderly people considering their actual needs, and door-to-door mental health education and mental hygiene services should be provided, while social activities beneficial to the elderly can also be organized in the community to improve their sense of social participation and alleviate depressive symptoms.

## 5 Limitations

The present study suffers from the following biases: the study is a cross-sectional study, and the results cannot be interpreted as causal; the data used in the study are cross-sectional data, and the results are only correlations between variables; most of the information on covariates was collected through questionnaires, and comorbidities were self-reported, with recall bias. In the future, additional attention should be paid to the content of the new questionnaire in the database, and the statistical yearbook data should be used to understand the current situation related to old depression in elderly patients with cardiovascular and cerebrovascular diseases, and longitudinal studies should be carried out and analyzed, in order to lay the foundation for a comprehensive understanding of the influencing factors of depressive symptoms in elderly patients with cardiovascular and cerebrovascular diseases.

## 6 Conclusions

With the continued aging of the population and the improvement in people’s living standards, more and more people were concerned about the mental health of the elderly. Depression, as one of the important indicators of mental health, was also receiving more and more widespread attention from society. In this study, the level of depression of elderly patients with cardiovascular disease was divided into three subgroups, with different characteristics between each subgroup. By analyzing the key influencing factors and their mechanism of influence on depression, this study could provide a basis for the effective prevention and treatment of depression in the elderly by the health sector and other related departments, provide a theoretical reference for improving the mental status of the elderly in China, and be of great significance for the realization of healthy aging.

## Data Availability

The raw data supporting the conclusions of this article will be made available by the authors, without undue reservation.
